# Ukrainian health care system and its chances for successful transition from Soviet legacies

**DOI:** 10.1186/s12992-018-0439-5

**Published:** 2018-11-23

**Authors:** Piotr Romaniuk, Tetyana Semigina

**Affiliations:** 1 School of Public Health, Department of Health Policy, Medical University of Silesia in Katowice, Ul. Piekarska 18, 41-902 Bytom, Poland; 2 Academy of Labour, Social Relations and Tourism, 3A, Kiltseva Doroga, Kyiv, 03187 Ukraine

**Keywords:** Ukraine, Health reform, Health system, Health transition

## Abstract

**Background:**

Ukraine, one of the largest states formed on the rubble of the Soviet Union, is widely perceived as a country that has lost its opportunities. Being devastated by corruption, it shows incapable to modernize and enter the economic path of sustainable growth. Similarly in the health care system no deeper reform efforts have been taken in the entire post-soviet period, leaving the system in bonds of ineffective solutions taken out of the Soviet era.

**Main body:**

The recent geopolitical and economic crisis seem to favor the introduction of radical solutions that might lead to a rapid change in the organizational paradigm of the economic system, as well as in health care in Ukraine. In this paper we aim to highlight the key features of the ongoing health reform in Ukraine, identify basic challenges for it, and assess rationality and feasibility of the reform. We found that the projected scope and schedule of changes in the Ukrainian health system give promising prognosis regarding its final effect.

**Conclusions:**

The final success of health reform in Ukraine is dependent on a number of factors, including the financial foundation arising of economic stability of the country, balance assurance between public and private spending for health and ability to eliminate the long-lasting practices, particularly when they are connected with activities of lobbying groups occupying particular positions in the health system. A consequence of actions taken by the political decision-makers in the longer perspective are also to highly determine the reform’s chances for success.

## Introduction

Ukraine is one of the largest states formed on the rubble of the Soviet Union. Its most recent history is marked by a series of dramatic events and drastic turmoils. Currently, Ukraine is widely perceived [1, 2] as a country that has lost its opportunities. Highly industrialized, with all the advantages allowing it to create also a modern agricultural system, it has become stagnant in the transformation process, making virtually no step forward for the entire period of transformation. The country is devastated by corruption [3–5], and all attempts to modernize it and push it to the economic path ensuring competitiveness in global markets are hampered by the interests of private individuals who have appropriated a large part of the economic system in the course of privatization process carried out in an unfair way. Hale and Orrtung [6] stressed that plutocratic «oligarchs,» and the economy patrimonialism have strong latent influence on the lack of reforms in Ukraine and constitute fundamental contextual challenges.

Currently, the country is characterized by considerable differences in the level of income and tensions between supporters of close ties with Russia, pro-Western forces and nationalists [7]. The armed conflict, or ‘hybrid war’, on the territory of the eastern regions of the country [8], the annexation of Crimea by Russia [9] and the internal tensions that we observe over the past few years, has deepening the difficult economic and social situation of the country.

As in the Ukrainian economy the changes occur in a slow and uncontrolled manner, similarly in the health care system no deeper reform efforts have been taken in the entire post-soviet period. As a result, from the legal standpoint the Ukrainian system is so far formally functioning in accordance with the organizational assumptions of the Semashko model, with central budgetary financing, lack of pro-effective solutions in the financing of medical services (global budgeting of health units), hierarchical organizational structure, and dominance of public sector [10]. In the context of transition, it is expected [11] that the European post-communist states would develop health insurance model, eradicate informal payments and transform the outdated highly centralized health care system. While the other post-communist countries undertook an effort to meet these expectations, the Ukrainian health system remained in a virtually unchanged form throughout the entire period after the collapse of the Soviet Union. Any changes that were applied, were actually apparent and inferior. The process of decisional decentralization was based on the structures of governmental administration, which made it superficial and only partial: there was a shift or responsibilities regarding the ownership over health care facilities to the level of local authorities, on a limited scale the private sector was allowed to participate in the health system, but outside of the scheme of public financing, based on direct patients’ out-of-the-pocket payments, mostly informal. Within the framework of public services provision system patients theoretically had the right to choose a doctor and facility. In practice, however, this was an apparent right, and the way how the system was constructed and acted treated their needs and rights without due attention [10].

At the same time in both these spheres – the economy, and the health system – activities have been undertaken in the last few years to stabilize the situation and bring Ukraine to the path of stable and long-term growth and normalization. The severe circumstances seem to favor the introduction of radical solutions that might lead to a rapid change in the organizational paradigm of the economic system, as well as in health care and other areas of State’s activity in the field of social policy [12, 13]. The introduction of pro-efficiency solutions seems to be a necessary factor, without which, in turn, increased financing of the health sector will not provide improvement on a scale that would be adequate to the social expectations, as well as would be in line with the assumptions of the decision-makers who decided to implement the reform [14].

In August, 2014 Ministry of Health of Ukraine initiated the development of National Strategy on Health Reform to revitalize and speed up the process of reforms in health sector through elaborating strategic approaches to improve the quality and access to health care and ensuring the mitigation of financial risks for population [15]. In 2016, the Cabinet of Ministries of Ukraine approved the Concept of Reforming of the Health Care Financing. In 2017, a few legislative documents adopted by the Verkhona Rada (Parliament) of Ukraine, as well the orders adopted by the Cabinet of Ministries of Ukraine has opened the process of the re-shaping the Ukrainian health care system providing a new approach to the financing of healthcare institutions and individual healthcare practitioners. The introduction of a new system on the primary level is planned for 2018, while the whole reform will be incrementally conducted until 2020.

In this paper our purpose is to highlight the key features of the ongoing health reform in Ukraine. The paper starts from needs assessment and problem analysis within the health care system of Ukraine. The next section of the paper reviews the previous attempts to deal with the existing problems and lessons from the past. Then the paper presents the key features of Ukrainian health system reform and identifies several challenges for it, each representing a possible stalemate between vested interests of the actors involved. Taken together, these locking-ins may block the course of reform in given direction thus increasing the tensions in the health system as a whole.

The paper is based on the adopted framework for ex ante policy analysis, with the focus on the feasibility and relevance of the policy implementation. The paper looks at whether the planned effects are desirable and achievable. We also present the background for the reform process, including analysis of the epidemiological status of Ukrainian population based on data from Health for All databases, data from the Health Index, and Ukraine surveys conducted in 2016 and 2017 by the Kyiv International Institute of Sociology. In this section we compare the selected indicators of the Ukrainian population health status with similar data for post-communist countries that has joined European Union. We took this group of countries as a contrasting benchmark, mainly because all of them has applied some sort of health reform during the period of post-communist transition, and usually they are being presented as examples of success of such transition in the global aspect. Desk-review of key documents on health care reforms in Ukraine has been a basis for policy analysis.

## Needs for changes in the Ukrainian health care system

A measure for a collapse in the Ukrainian health system is the epidemiological situation of the country’s population, as well as changes that have occurred in its health status since the end of the Soviet era [14, 16, 17]. The *Health for All* database [18] reveals that mortality due to cardiovascular diseases in 1990 amounted to 589.03 deaths per 100,000 inhabitants in Ukraine. In the later period it has increased, amounting to 634.59 deaths per 100,000 inhabitants in 2015. For comparison, in the post-communist countries that became Member States of the European Union (Bulgaria, Croatia, Czech Republic, Estonia, Hungary, Latvia, Lithuania, Poland, Romania, Slovakia, Slovenia) the average value of this measure amounted to 625.73 deaths per 100,000 population in 1990, and by 2015 it has decreased to the level of 362.5 deaths per 100,000 population. Thus, in this group of countries we observed an improvement by 42%, while in Ukraine in the same period the mortality has increased by almost 8%. What’s important, in the initial period mortality in Ukraine has been lower than in most of the other countries included in the comparison group. Additionally, in some of them in the first half of 90’s, and in some others – in the second half of the first decade of transformation, there was the increase of the mortality rate observed. These are the cases of Romania, Latvia, Bulgaria and Estonia. Nonetheless, all of them experienced clear and generally regular drop in subsequent years, while in case of Ukraine there is the increase observable up to 2005, and only in the recent years the country notice some improvement, although having the result worse than any of the comparison countries individually. The negative trend is even more evident in case of data for ischaemic heart disease, where in the Ukraine over the period considered the mortality increased by over 36%, although in the group of “new” EU Member States at the same time similar was the percentage of decrease in the average value of mortality rate.

In the Ukrainian health system the failure of its efficiency, both in terms of treatment, and preventive interventions, is also well illustrated by other epidemiological data. Mortality due to infectious and parasitic diseases between 1990 and 2015 increased from 11.78 to 21.67 deaths per 100,000 population. In the adopted reference group at the same time there was a decrease from the level of 8.76 to 7 deaths per 100,000 population. This difference is even more striking in case of tuberculosis, where in 2015 mortality in Ukraine amounted to 9.89 cases per 100,000 population, while in the reference group – 1.77 deaths per 100,000 population.

Measures that are highly susceptible to systemic changes also illustrate significant negligence of the Ukrainian system. Infant mortality rate in the period 1990–2015 decreased from 16.6 to 8.1 deaths per every 1000 live births, however, for the group of post-communist countries that joined the European Union, there was significantly higher drop. In 1990 the average value of the factor was similar there to the one in Ukraine (16.05 deaths per 1000 live births), while in 2015 it has decreased to an average value of 4.85 deaths per 1000 live births. The situation is similar in case of maternal mortality, where in Ukraine in the discussed period the value of the factor dropped from 32.41 deaths to 14.81 deaths per 100,000 live births (data for 2014), which means a decrease of over 54%. At the same time in the reference group the average value of the factor decreased from 28.68 to 7.56 deaths per 100,000 live births, which means an improvement by nearly 74%. Data on the life expectancy show that in case of Ukraine there was some improvement: from 70.53 years in 1990 to 72.5 years in 2015, but it is significantly worse than in compared group of countries, where the initial average value in 1990 was similar like in Ukraine (70.84 years), but the life expectancy has increased to 76.87 years in 2015. Furthermore, the group of “new” EU Member States managed to reduce the distance to “old” EU Member States in this respect – the difference decreased from 5.64 years to 5.05 years, while for Ukraine this distance has increased from the initial level 5.95 years in 1990 to 9.42 years in 2015.

Finally, in Ukraine we can observe a substantial drop in the proportion of infants vaccination. For example, the percentage of children vaccinated against measles, although yet in the previous decade exceeded 90%, in recent years has drastically dropped to the level of 42% in 2016. A similar situation applies to vaccination against tuberculosis – from a level exceeding 90% to as much as 39% in 2015.

Ukraine has over 2200 hospitals and over 400,000 hospital beds (5,22 hospitals and 890,7 beds per 100,000 population) in the public sector. In per capita terms, this is more than in EU countries. But the facilities have outdated equipment and very few are able to provide complex care [10]. Until now, Ukraine’s healthcare financing mechanism was based on a general taxation system, in which expenditures were split between state (national) and regional budgets. In 2015, public financing of the healthcare system reached 71 billion UAH (around 3.2 billion USD) or around 3.5% of GDP, while total, public and private, expenditures constitute 7.4% of GDP. Due to low value of Ukrainian GDP, this expenditures, although they may seem quite high in relative terms, translate into severely low level of per capita spendings, amounting to less than USD 300 in 2012. The primary health care receive about 10% of health care financing, and the major share of costs goes to highly specialized care [15].

In 2005–2015, total volume of financing increased by more than five times. However, such boost may be explained mostly by currency devaluation: the mean annual exchange rate for one USD rose from 5.13 UAH in 2005 to 21.85 UAH in 2015 [19]. It is worth to mention that the distribution of funds for the health care is carried out according to the existing infrastructure, and not to the real needs defined by the structure and levels of morbidity of Ukrainian population [20]. Most recent results of the national household surveys *Health Index. Ukraine* proves that the access to medicines remains a significant problem for Ukrainian population. Households spent nearly 11% of the average total expenditures to purchase drugs. According to the other household survey, 92% of the population is afraid of getting into financial difficulties and of catastrophic expenditures in case of illness [21]. There is evidence for limited access to diverse categories of health services. Studies revealed that up to 75% of people with common mental disoders and alcohol use disoders in Ukraine do not access care. Barriers to care include stigma and shame, fear of psychiatry and lack of trust in the health system, but also they derive from system deficiencies, like lack of information and awareness, high cost of treatment, fear of having a public record as being diagnosed with mental illness, and geographical distance [22].

Aggregated data on the health system outcomes also show relatively poor progress in the last three decades, as well as significantly worse results compared to the average for the entire group of post-communist countries [23, 24].

## The previous course of health reform processes

During the period after the fall of communism, there was a systematic increase in financial burdens on the side of the patients. The system formally declares a very wide and universal range of services guaranteed to patients under public funding, but in practice an ineffective structure and very limited financial resources allocated for this purpose make it a purely legal right. In recent years, private spendings have reached the level of 45% of total expenditure on health care [10]. A huge part of the funds from this source is absorbed by the pharmaceutical sector, which is practically completely deprived of public financing, and at the same time has not been provided with solutions to rationalize expenditures [25]. As a result, the burden on patients on this account is extraordinarily large. Although in 1996, the government took an effort to normalize the situation and the official rates of fees charged to patients for the provision of services not covered by public guarantees had been established [26], in practice this mechanism was a fiction, and the boundary between paid and free benefits remains highly unclear. An additional complicating factor is the widespread mechanism of informal payments, which is a substitute for missing official mechanisms that would rationalize the system and ensure its financial efficiency. Funds allocated to health through this channel are a very heavy burden on patients’ home budgets. What should be emphasized, is that all this takes place in conditions of a relatively high level of total expenditure on health, amounting to 7.6% of GDP in 2012 [10, 27]. This is an evident testimony of far-reaching inefficiencies in spending funds in the health system.

In the previous decade, in order to improve the functioning of the system, public authorities undertook certain activities aimed at the increase of popularity of additional health insurance – both in commercial form, and through the so-called sickness funds, whose role is analogous to commercial insurers, with the only difference that they act as a non-profit basis [28]. The popularity of these solutions, however, remains low, not exceeding 2–3% of the country’s population in total for both formulas. Following the traditional solutions of the Semashko model, the Ukrainian system is also characterized by a number of parallel system solutions for some groups of the population. Such systems absorb a large part of the public funds allocated to health care, all the more hindering the implementation of equal and uniform solutions. Some of these parallel systems were created already in the period after the fall of communism, referring somewhat to the classical Bismarckian solutions. In such categories the insurance system for employees in the transport sector might be considered [10, 29].

The Ukrainian system is undoubtedly one of the weakest among post-communist European states. It is characterized by organizational and financial inefficiency, inadequacy to the population’s health needs and the lack of deeper reform efforts throughout the post-communist transition period. All those that took place and were aimed at modernizing the existing solutions (introducing the model of family medicine in primary care, changing the methods of financing services, introducing additional financing mechanisms) were purely initial in nature, being introduced only in some areas of the country, or as a kind of trial mechanism. All this is reflected in the low level of response to the health needs of the individuals and groups, which in turn translates into poor health status of the country’s population. The system is characterized by low ratings not only in the sphere of health outcomes, but also in terms of access to services, where one of the main barriers is the financial issue, equality and equity, as well as lack of implementation of activities aimed at positive stimulation of the health potential of the country’s population. The difficult political and economic situation of the country are additional factors that produce obstacles to effectively improve the efficiency of the system. The low level of GDP does not provide adequate financial support, and the geopolitical and economic turbulence of recent years have additionally led, as should be supposed, to the collapse of the sector of some services.

## The projected scope of reforms

The most recent political revolution in Ukraine (2014) has once again opened the chance to implement a deep modernization project of the Ukrainian state. The will to conduct such an ambitious project was clearly declared by political leaders, and one of the symptoms of such engagement was the desire to replace decision-making elites. The way to achieve this aim was intensive recruitment of individuals from other countries for the managerial positions in public administration and other centrally managed entities.

**Fig. 1:**
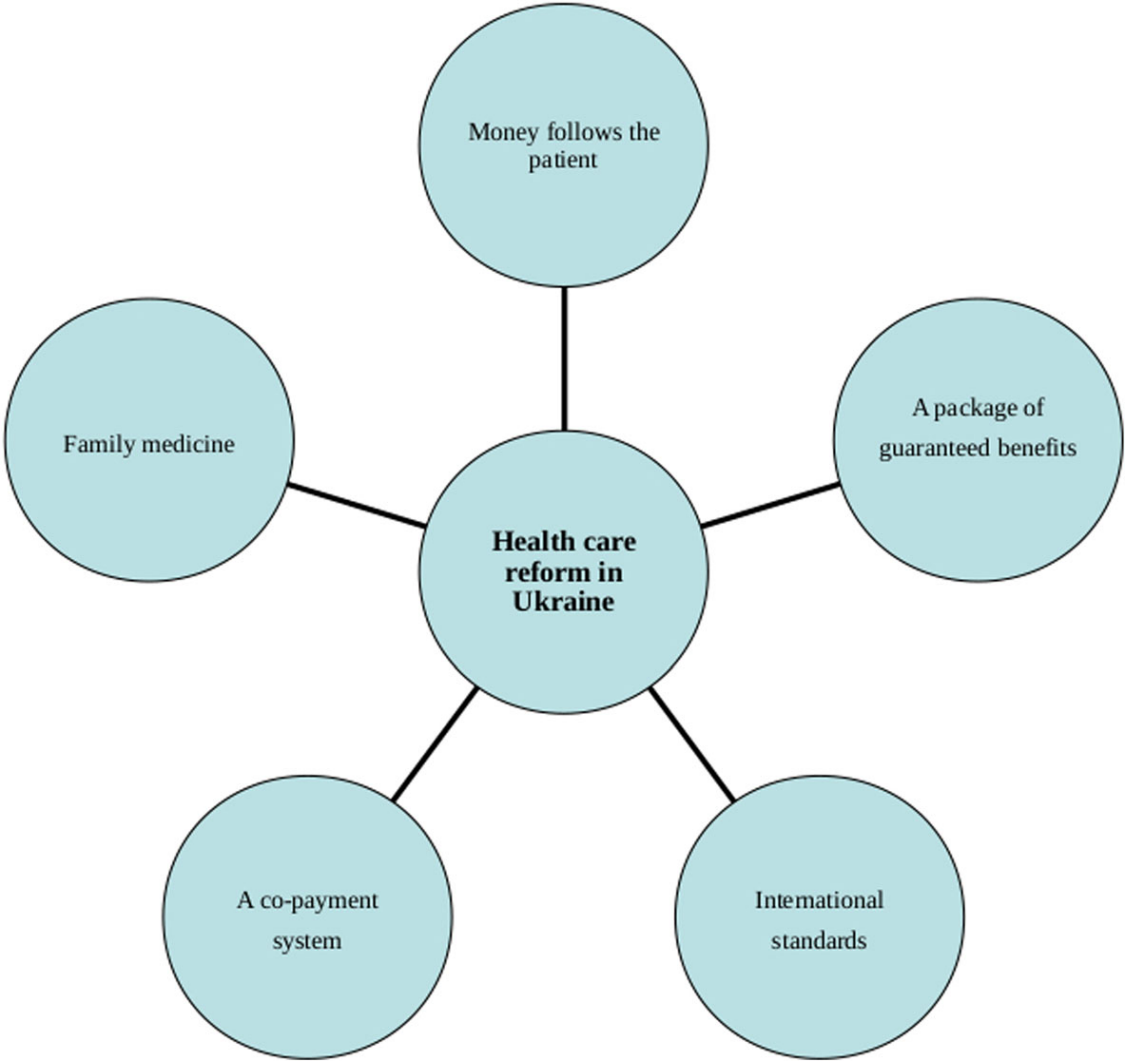
The Ministry of Health of Ukraine vision on health care reforms (2015). Source: own

This reforming enthusiasm also applies to the anachronistic structure of the health system. In 2015, as an initial step of reforms, Ukraine handed procurement of vaccines, medicines, and medical equipment to international agencies: the United Nations Children’s Fund (UNICEF), United Nations Development Programme (UNDP), and the UK nonprofit entity Crown Agents. In the same year, the Ministry of Health lead by Georgia-born *Alexander Kvitashvili* and then by USA-born Ulyana Suprun has proposed a reform package based on five main pillars [30] (see Fig. 1).

The first pillar of the Ministry’s concept was about reorganization of the system of financing health services, where the main change is to shift the previously applied model of financing facilities to financing the patient’s needs, in accordance with the popular wording “money follows the patient”. This is a change which should be seen as an attempt to apply solutions previously implemented in other post-communist countries. Its main assumptions imply the need to change the relationship between the payer and entities that deliver the health services, where the latter are forced to adapt to the conditions proposed by the payer. These, according to assumptions, should be derived from real population’s needs. In other words, the range of services provided by the providers and their quantity should be derived from diagnosed health needs, as well as a resultant of consumer decisions regarding the choice of facility, which in turn opens the Ukrainian system to internal competition [30, 31].

The second Ministry’s pillar was the introduction of the medical specialization in the field of family medicine. Family doctor on the one hand should become the main coordinator of the patient’s health care process, and on the other hand – will be subject to patient’s choice on an individual decision basis, in contrast to the current construction, which is based on the reionisation system originating from the Soviet era. In addition, the financing of the family doctor is to be based on the per capita rate, where the doctor’s income depends on the number of patients declared to be under his care. This is also a solution referring to projects previously implemented in other post-communist countries [31].

The next pillar within the Ministry’s concept was that the State undertakes to clearly define the scope of its financial responsibility in health care, which should be treated as tantamount to introducing a package of guaranteed benefits. According to the declaration of the Ukrainian Ministry of Health, the full range of reimbursement is supposed to cover family medical services, palliative care, emergency medicine, and pharmaceutical treatment for cardiovascular diseases, bronchial asthma and type 2 diabetes. Finally, the full scope of State’s responsibility is also to cover childbirths, rare diseases and oncological treatment. Such solutions were expected to help to clarify the financial responsibility of the state, but also to limit the phenomenon of informal payments, commonly appearing in the health system [31].

In other areas, not included in the package of fully guaranteed service, a co-payment system is projected to be implemented, including tariffs approved by the State and remaining within its scope of regulation, and the complementary insurance system. At the same time, a certain range of services is to be completely excluded from the range of publicly financed benefits (eg aesthetic medicine). And this idea constituted the forth pillar of the Ministry’s concept [31].

The last key element of the Ministry’s reform vision referred to the characteristics and quality of services provided, where, most importantly, physicians operating within the system are to be obliged to implement procedures in accordance with current international standards, instead of the present ones, which are outdated and inadequate to the current state of medical knowledge. The procedures applied hitherto are also susceptible to fraud and corruption – treatment of certain diseases with expensive and inadequate medicines results not so much from the actual health needs, but more from informal arrangements that give financial benefits to specific persons (including government officials deciding on the application of a given procedure). The problem is also related to the reform of the medical education, first of all by providing access to the most recent achievements in the field of medical sciences, but also by eliminating the corrupted system of granting the right to practice the medical profession and providing an objective, external system of competence assessment of graduates [31].

Among the assumptions related to the systemic changes the Ukrainian government plans to implement, there are also ones located cross-sectionally among the above-mentioned strategic goals. One of them is an assumed change of the distribution model and the implementation of vaccination. Currently the supply is based on ad hoc purchases, which is supposed to be replaced with a three-year planning system. On the one hand, such a solution should increase the availability of vaccines for patients, and on the other – eliminate abuses related to purchasing vaccines. The Ministry of Health plans also to modernize facilities providing health services and apply widely computerization of the health system, which should include the possibility for patients to use on-line registration. In addition, Ukraine declares its will to implement the public health perspective in policy evaluation applied in other sectors of State’s activity, including the area of fiscal policy and using its instruments to stimulate health behaviors [31].

The changes in the organization of the system of service provision require to reorganize also the area of management and control over the financial resources. The Ukrainian government has taken intensive action to achieve this goal. In 2018 the works started to implement new organizational scheme with the main focus on the central body, the National Health Service of Ukraine (NSHU). The new institution is supposed eventually to act as a public agency responsible for contracting health service providers. Simultaneously, the process of implementation of a new financing scheme has been initiated, in the first place at the level of primary health care. Except for the change of money flows, the reform allows also private providers to be contracted by public payer. The use of new financial mechanisms is planned to come into force in the area of specialist treatment in 2019, whereas in 2020 the implementation of solutions for guaranteed access to services is supposed to take place. Some ad hoc investment are also being applied to improve the supply of medicines and vaccines, as well to ensure general improvement of the infrastructure of service provision (Ministry of Health of Ukraine, 2018).

This Ministry’s concept had been supported by the numerous international organizations, including WHO, UNDP, USAID etc. These organizations have been constantly pressing for faster reform in Ukraine [32]. At the same time, not all ideas of the Ministry of Health were accepted by the Ukrainian politicians, especially by the opposition groups. The opponents of the changes, including many members of the Parliament and media, had built up their messages around the idea that the reform could leave the poorest with no healthcare. The campaign was also supported by the medical lobbyist groups who may want to preserve their control over the flows of informal payments and corrupted drug procurement system [33].

On October 19, 2017 the Ukrainian Parliament finally passed a law that will start medical reform in Ukraine [34]. Right after passing the law on financing healthcare, the Ukrainian parliament approved two other related bills: one is aimed to provide people in remote areas with access to medical services, including telemedicine [35], and another that amends the financial code of Ukraine [36]. The law stipulates a series of changes to be made by 2020, including the introduction of an insurance system financed by the government, and a mechanism to allow patients to choose doctors and hospitals themselves. A nationwide e-system for tracking patients’ health history will be implemented, starting in 2019. In addition, the new system should reduce the problem of self-treatment in Ukraine — the patient will be incentivized to seek treatment from a doctor, since reimbursement will only be provided upon the prescription of the healthcare professional. However, the Ukrainian parliament didn’t cast votes in favor of co-payments for health care services, as this idea is highly unpopular among patients and medical staff [37].

## Feasibility of the reform implementation

The described actions are aimed at satisfying the most urgent deficits and needs in the health system. Taking them first, from the point of view of reform process rationale, seems to be highly justified. On the one hand, it allows to satisfy the most urgent populations’ health needs and expectations in a relatively short time. Since in the current systemic realities these needs and expectations are treated in a far inadequate way, such a way of acting gives an opportunity to ensure social support for the reform process and thus eliminate at least some of the barriers limiting the chances of its successful completion.

One of the core ideas of Ukrainian transition from the Soviet legacies is that the existing “pay-per-bed” approach will be substituted by a “money follows the patient” principle. It means that instead of funding specific hospitals, the state will pay for the number of patients referring to the specific provider. The proposed direction of the reform should be considered as justified by the practical premises and provides a good foundation for building a pro-effective formal and structural base in the system [38–40].

However, there is a number of threats that may ultimately blur the essence of the reform, or to a significant extent minimize its positive effect and induce a negative perception among the society and health professionals. Firstly, the reorientation of the financing model and its leaning on the consumer choices made by patients, it should have a positive effect in terms of improving the quality of services provided. Nonetheless, in a situation, where there are limited financial resources designed for health care, the shift towards such model will have to involve implementation of solutions that rationalize the demand, and thus it may cause the extended waiting time for certain categories of services. Limitation of access yet at the level of systemic decisions may, in turn, lead to an increase in public dissatisfaction, as well as may imply the development of alternative mechanisms for financing excessive demand. While the implementation of projected solutions at the level of primary health care is unlikely to produce this kind of effect, in case of specialist services and some other services such a risk exist in reality, as evidenced by the experience of other countries [41–44]. Finally, there is a real risk of inadequate distribution of funds for services, where the increased funding will result from pressure of the entities operating on the demand side, as well as certain consumer decisions of patients generated by reasons other than the actual health need. The final success in implementing the discussed solutions in a strong manner depends therefore on increasing the scope of publicly covered services. Additionally it would require to simultaneously implement system-wide solutions aimed at the identification of demand and subsequently – supply planning for a specific type of services. Maps of health needs might be an example of potentially useful solutions of that kind [45, 46].

It is worth to stress that the change in the financing model at the level of primary care itself is a relatively simple solution to be implemented, connected with a number of possible advantages, namely the stimulation of internal competition, improvement of the quality of services and rigorous cost control [47]. The more difficult challenge is to realistically strengthen the systemic role of primary health care. This difficulty results from several threats and barriers. First, strengthening the role of the family doctor is associated with the need to thoroughly rebuild the educational system, and to establish a system of incentives for the family doctors to continuously improve their competences, and to ensure the professional and financial attractiveness of primary care practice. Secondly, it is necessary to systematically and intensively support the primary health care sector in order to eliminate the prevailing habits among the medical specialists, as well as patients, who use to perceive this sector of treatment in terms of inferiority. Both of the issues mentioned remain in close relationship with each other. While the systemic need would lead to increasing the burden put on the primary care sector, the lack of well trained family medicine specialist staff may be an effective barrier in this respect [48, 49]. Another possible difficulty is the appropriate shaping of financial relations between the sector of primary health care and higher levels of health care. Implementation of financial responsibility of primary care physicians for services provided on the basis of their referral may result in unjustified restriction of access to diagnostic and specialist services. This limitation is to some extent expected and necessary to eliminate excessive demand, however, it may not be considered in terms of a barrier to satisfying health needs only when providing an adequate level of competence and commitment of the family physicians themselves. In turn, limiting the role of the family doctor’s responsibility may result in shifting costs to other healthcare sectors, which subsequently may make apparent the assumed savings resulting from the changed funding formula, and so may become the ability to cover them. Also under these circumstances, the availability of specialist services may be limited because of the lack of systemic ability to cover the costs of growing demand [50–52].

The implementation of a package of guaranteed benefits, in particular if constructed positively, is considered to be an important and necessary condition for ordering the financial responsibility of the state in health care, as well as for regulating the financial flows in the system in general [53, 54]. However, the difficulty in implementing such solutions is the ability of reaching a consensus on the range of public responsibility for access to services. If the package is defined too extensively, it exposes the system to financial shortages and creates a risk of State’s responsibility to become ostensible, and the access to services purely virtual. In the absence of appropriate funding streams, shortages have to be covered by private funds, or result with the extending waiting time for guaranteed services [55]. On the other hand, these private funds, remaining out of control in terms of the rationality of expenditures, only to a limited extent contribute to the improvement of the epidemiological status of the population [23]. Actually, this is the state of things Ukraine is struggling so far. In this context the liquidation of a specific type of fiction operating under the present conditions is certainly a rational action.

In the process of health care reform implementation the difficulties resulting from long-lasting practices must be considered. These practices are strongly inscribed in the systemic tradition and in the habits of its participants. The key barrier to the implementation of the above postulate may be resistance from the interest groups controlling the procedures of granting rights to practice the profession, who used to enjoy concrete financial and prestigious benefits from this fact. They are at the same time influential enough to be able to block changes unfavorable for their current status. The petrification of the education schemes applied years ago, and the lack of extensive interaction with international scientific communities, may constitute a serious obstacle to the modernization of the education process of medical cadre. Unlike in most of the previous cases, the main barrier in the process of implementing this part of the reform is to a lesser extent of financial nature, and more – resulting from mental factors.

Political issues are also of importance, related to the possible activation of lobbying groups. There is a possibility that the process of achieving the assumed goal in this dimension may take enormously long time or could be revised after the next Presidential elections in 2019. While adopted in 2017 health care reform has strong pro-market associations, the Ukrainian politics is considered to be a rather populist and leftist, as concluded by White (2010) [56], who pointed out that the values of the Soviet period remain intact, same as the political ideas of the left parties.

The final success of the actions taken will depend on a number of factors, not all of which are control-sensitive modifiable factors, subject to public authority stimulation. This refers to the geopolitical context or the global economic situation, as well as the possible resistance of the society, which may be unwilling to accept the implementation the reform program. This is even more probable in case of interest groups, for whom maintaining the status quo in a given area of change is beneficial from the point of view of satisfying their own needs. Despite the public support for the eradication of informal payments, there are population groups who favor their existence and this should be taken into account in policy-making [57]. If the health sector itself is taken into account, the final reform success depends to a large extent on changes in the economic sphere, where possible reform measures concerning the health system may bring the final effect unsatisfactory in case of lacking appropriate financial foundation resulting from economic prosperity [23].

## Concluding remarks

Both the planned scope of changes in the Ukrainian health system, as well as the way it is scheduled, along with consistency in the implementation of subsequent reform stages, give promising prognosis regarding the final effect of the reform. An important feature of the government’s project is a focus on satisfying the most urgent and most pressing needs identified in the system, which are related to the access to services and medications supply. It goes in line with views on distribution of health care and using the solidarity type of social policy instruments [58] and at the same time reflects changes of social policies in post-communist states where state-planned economy gradually transforms to the capitalist system [59, 60].

The current health care reform assumes implementation of solutions that replicate those that have been empirically verified based on the experience of other countries, especially those that have followed a similar path of transformation before [61–67]. The reform project seems to be rationally laid out in time, with supposed finalization within a perspective of several years. However, the actual success of the undertaken activities depends on a number of additional factors, both external and related to the assumptions of the reform. In particular, a special attention should be paid to macroeconomic conditions and provision of an adequate resources for health services. In this context the level of health spendings related to GDP is of importance, but first of all the expenditures counted in absolute amounts matters, since the impact of this factor on the health system outcomes is definitely more strongly noticeable [23]. In other words, the success of the reform in health care is directly dependent on the success in the transformation and growth of the Ukrainian economy.

The second key aspect of the system deficiencies that are money-related is the scale of State’s involvement in health care, where, as mentioned, a large scale of financial burden of health expenditures lay on the side of patients themselves, which constitutes a serious barrier limiting the possibility of receiving appropriate treatment. As such, this problem may be a strong limitation to ability of achieving the assumed aims of the reform in the epidemiological dimension. The generally low level of affluence of the Ukrainian population may be also a factor limiting the popularity of proposed complementary instruments of protection, which will finally cause the real reform only apparent.

Except of the financial aspects, there are other factors as well, which may constitute a threat to the final success of the reform. One of them is the late moment of taking corrective actions, which resulted with consolidation of different kinds of patologized processes substitutive to the inefficient formal public system. This substitutive instruments of regulating access to services and health financing has produced a set of habits observable on the side of providers as well as patients. These habits may turn out to be difficult to be eliminated, particularly when they affect vital interests of influential lobbying. For this reason the reform leaders must take into account the possibility that the process of change in the short term will have limited effect, and full implementation of its objectives may be a long-term process. Even in this long-term perspective the final success rely on the full consequence of actions taken by the political decision-making center in implementation of the chosen reform path. Failures of previous reform attempts, which were largely based on similarly defined priorities, can be seen as additional evidence of the uncertainty of the final outcome and its susceptibility to undesirable influences of disruptive factors.
